# Gene-regulatory network analysis of ankylosing spondylitis with a single-cell chromatin accessible assay

**DOI:** 10.1038/s41598-020-76574-5

**Published:** 2020-11-10

**Authors:** Haiyan Yu, Hongwei Wu, Fengping Zheng, Chengxin Zhu, Lianghong Yin, Weier Dai, Dongzhou Liu, Donge Tang, Xiaoping Hong, Yong Dai

**Affiliations:** 1 Clinical Medical Research Center, The Second Clinical Medical College of Jinan University (Shenzhen People’s Hospital), Shenzhen, 518020 Guangdong China; 2 Integrated Chinese and Western Medicine Postdoctoral Research Station, Jinan University, Guangzhou, 510632 China; 3 Department of Nephrology, The First Affiliated Hospital of Jinan University, Guangzhou, 510632 Guangdong China; 4 College of Natural Science, University of Texas at Austin, Austin, TX 78721 USA

**Keywords:** Genetics, Immunology, Diseases, Pathogenesis

## Abstract

A detailed understanding of the gene-regulatory network in ankylosing spondylitis (AS) is vital for elucidating the mechanisms of AS pathogenesis. Assaying transposase-accessible chromatin in single cell sequencing (scATAC-seq) is a suitable method for revealing such networks. Thus, scATAC-seq was applied to define the landscape of active regulatory DNA in AS. As a result, there was a significant change in the percent of CD8+ T cells in PBMCs, and 37 differentially accessible transcription factor (TF) motifs were identified. T cells, monocytes-1 and dendritic cells were found to be crucial for the IL-17 signaling pathway and TNF signaling pathway, since they had 73 potential target genes regulated by 8 TF motifs with decreased accessibility in AS. Moreover, natural killer cells were involved in AS by increasing the accessibility to TF motifs TEAD1 and JUN to induce cytokine-cytokine receptor interactions. In addition, CD4+ T cells and CD8+ T cells may be vital for altering host immune functions through increasing the accessibility of TF motifs NR1H4 and OLIG (OLIGI and OLIG2), respectively. These results explain clear gene regulatory variation in PBMCs from AS patients, providing a foundational framework for the study of personal regulomes and delivering insights into epigenetic therapy.

## Introduction

Ankylosing spondylitis (AS) is a chronic, inflammatory, rheumatic disease that predominantly affects the axial skeleton of young men^1^. Consequently, it causes characteristic inflammatory back pain, leads to structural and functional impairments and decreases the quality of life^2^. Various immune cells have been reported to play important roles in the initiation, progression and regulation of AS. For example, dendritic cells (DCs) are involved in Th17 immune responses, which are associated with manifestations of AS^3^. Natural killer (NK) cells can recognize HLA class I through the expression of genes named killer immunoglobulin-like receptors (KIRs). Interestingly, this recognition determines NK cell functions, one of which is recruiting other immune cells, resulting in an excessive immune state in AS^4^. T cells are able to mediate inflammatory responses by secreting inflammatory cytokines in AS, and B cells are reported to have a positive correlation with the Bath Ankylosing Spondylitis Disease Activity Index (BASDAI) in AS^3^. However, the number of these immune cell subsets and their roles in the pathogenesis of AS are still debatable. The precise etiology of AS remains unclear, which includes a lack of knowledge about the cell-type-specific regulatory program in the pathogenesis of AS.

Recently, assaying transposase-accessible chromatin in single cell sequencing (scATAC-seq) was devolved to map open chromatin regions and identify regulatory regions^5^. This method is not only simple and sensitive but also capable of recognizing different cell types, including subtle and rare cell subtypes, and it can reveal cell-type-specific regulatory regions and explore related transcription factors (TFs). Moreover, some recent studies have displayed the power of scATAC-seq for understanding regulatory principles. For example, the single-cell chromatin assay has successfully mapped the regulatory landscape of adult mouse tissues, characterized 85 distinct chromatin patterns, annotated key regulators in diverse mammalian cell types and identified cell types underlying common human traits and diseases^6^. Another group used scATAC-seq to study the mouse forebrain at eight developmental stages, and they found cell-type-specific transcriptional regulation and provided insight into forebrain development^7^. In addition, the application of scATAC-seq has identified cell-type-specific cis- and trans-regulatory elements without using antibodies and reconstructed trajectories of cellular differentiation in human blood; in addition, this method has disclosed regulatory networks in basal cell carcinoma^8^.

Notably, chromatin accessibility plays important roles in genome stability and gene regulation. As changes in chromatin accessibility patterns may alter the accessibility of key proteins to regulatory regions of the genome, these patterns are emerging as essential component of human diseases^9^. Using scATAC-seq to profile accessible chromatin of AS is a promising avenue for unveiling important cell subsets involved in AS pathogenesis without bias, and this process can explain how the chromatin regulatory elements govern transcription in each cell type. Thus, we take advantage of scATAC-seq to assess the landscape of open chromatin regions of peripheral blood mononuclear cells (PBMCs) from 9 AS patients and 12 healthy controls. Through analysis of scATAC-seq data, we performed cell identification without using antibodies. Then, we investigated novel and rare cell populations and evaluated the changes in immune cell numbers in AS patients compared to healthy controls. Finally, we analyzed cell-type-specific regulatory patterns. These results provided mechanistic insights into AS-associated pathogenesis.

## Results

### Benchmarking analysis of scATAC-seq

PBMCs from both AS patients (AS_PBMC) and healthy controls (NC_PBMC) were obtained and analyzed by scATAC-seq. In our study, patients treated with immune suppressants such as non-steroidal anti-inflammatory drugs (NSAIDs), corticosteroids (CS), methotrexate (MTX) and sulfasalazine (SSZ) within 3 months before sample collection were excluded (Table 1). After quality control was performed, 217,542,359 cleaned, paired-end reads from the AS_PBMC data and 222,111,738 paired-end reads from the NC_PBMC data were obtained. A total of 5,988,120 peaks of DNA accessibility from AS_PBMC library and 5,950,637 peaks from NC_PBMC library were identified. At single-cell resolution, approximately fourfold enrichment of fragments proximal to TSSs (relative to distal regions) from the AS_PBMC data were observed, and a similarly assessed fivefold enrichment from the NC_PBMC data were observed; these data reflect a high fraction of fragments captured within open rather than closed chromatin. The AS_PBMC library was sequenced at an average depth of 26,084 raw reads per cell, generating chromatin accessibility profiles for 8340 cells with a median of 6190 unique fragments per cell. Meanwhile, the NC_PBMC library contained 26,464 raw reads per cell, producing profiles for 8393 cells with a median of 5344 unique fragments per cell. In the AS_PBMC library, 19.12% of the fragments were assigned to TSSs, 13.01% were mapped to enhancer regions, 11.77% were linked to promoter regions, 22.36% were in nucleosome-free regions, 70.60% were mapped to a single nucleosome, and the fraction of total read pairs mapped confidently to the genome was 86.59%. In the NC_PBMC library, the related percentages were 21.57%, 13.25%, 14.29%, 23.71%, 69.04% and 86.77%, respectively.

**Table 1:** Clinical features of patients with AS and NC studied for scATAC-seq experiments.

Clinical characteristic (number of samples)	AS (n = 9)	HC ( n = 12)
Age (years)*	37 ± 6	34 ± 9
Sex, Female/Male	7/2	6/6
ESR(mm/hr)*	35 ± 38	NA
CRP(mg/l)*	18 ± 21	NA
Inflammatory back pain	8/9	NA
HLA-B27 positivity	7/9	NA
Schober test (cm)*	1.5 ± 1.4	NA
Hand to floor distance (cm)*	16.5 ± 16.5	NA
Chest expansion (cm)*	2.6 ± 2.0	NA

AS, ankylosing spondylitis; NC, healthy controls; PBMC, Peripheral blood mononuclear cells; NA, not applicable; ESR, Erythrocyte sedimentation rate; CRP, C-reactive protein; BASDAI, Bath AS disease activity index.

*Mean ± standard deviation.

### Cell-type-specific clustering of PBMCs

Clustering was performed with Cell Ranger ATAC pipeline, and 9 clusters were obtained for both AS_PBMC and NC_PBMC libraries after tSNE analysis. Following aggregating reads from all cells within a cluster to form ‘pseudobulk’ accessibility profiles and examining chromatin accessibility at known marker loci, 7 different functional cell types were recognized and annotated: NK cells (cluster 3), monocytes (cluster 4), memory CD4+ T cells (cluster 5 and cluster 8), CD8+ T cells (cluster 6), B cells (cluster 7) and DCs (cluster 9) (Supplementary Fig. S1). Meanwhile, none of the marker loci were significantly located in cluster 1 and cluster 2, and differential TF motifs between AS_PBMC and NC_PBMC libraries were hardly observed in these clusters. Since lymphocytes, monocytes and DCs were the focus of our research, cells from the relevant 7 annotated clusters were selected for reclustering and subsequent analysis, which included 4960 cells from the AS_PBMC library and 4094 cells from the NC_PBMC library.

**Figure 1:**
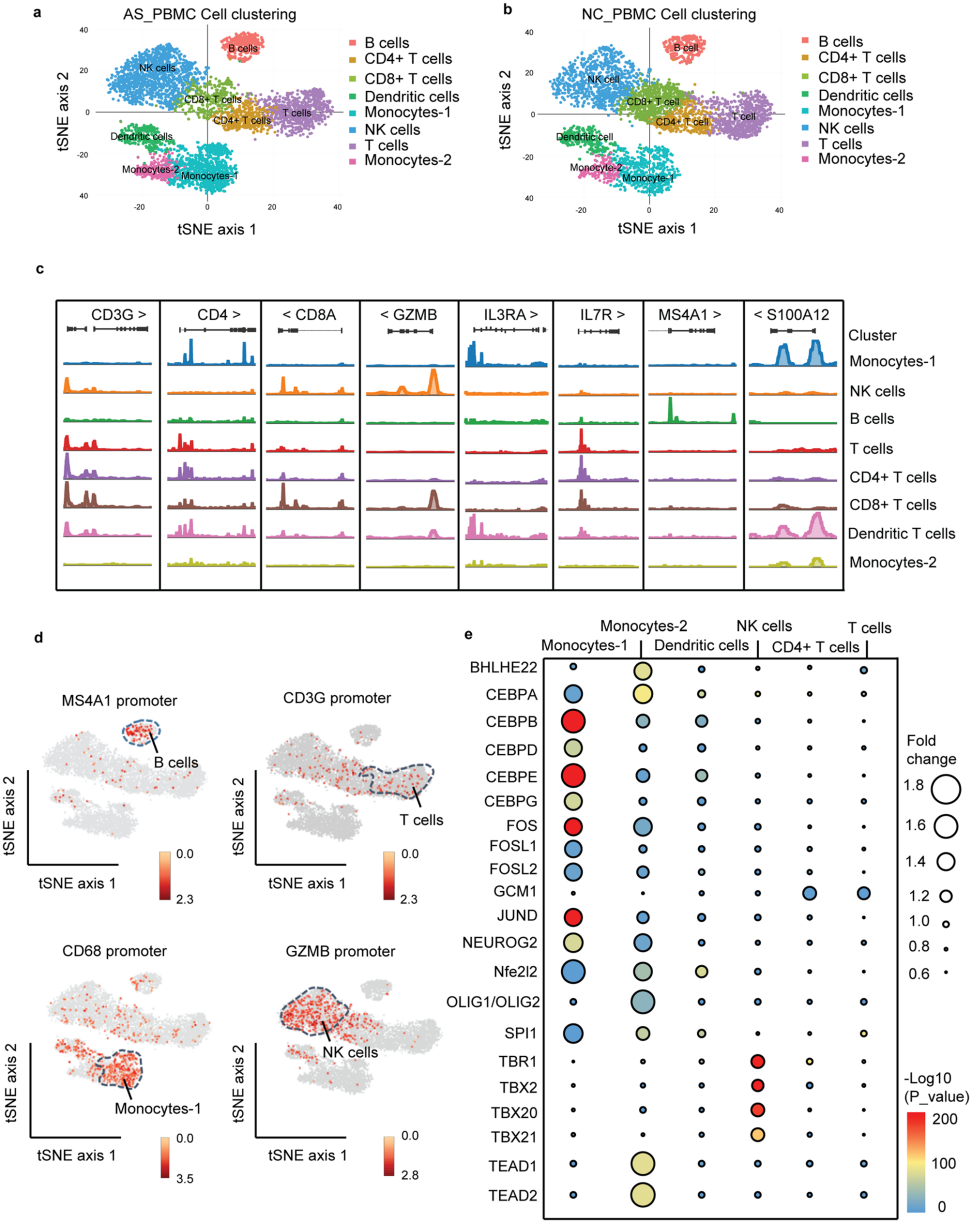
Cell-type-specific clustering of human PBMCs according to scATAC-seq. (**a**) Schematic of cell types in AS_PBMC group; (**b**) Schematic of cell types in NC_PBMC group; (**c**) Open chromatin signals for each cluster at several marker gene loci; (**d**) tSNE visualization of deviations in accessibility at marker gene promoters across the 8 clusters; (**e**) Heatmap representation of log twofold change in the 579 variable TF motifs (rows) across all scATAC-seq clusters (columns). PBMCs, peripheral blood mononuclear cells; scATAC-seq, assaying transposase-accessible chromatin in single cell sequencing; AS_PBMC, PBMCs from patients with ankylosing spondylitis (AS); NC_PBMC, PBMCs from healthy controls; NK cells, natural killer cells; TF, transcription factor.

As a result, 8 clusters were obtained and identified: NK cells (cluster 1), monocytes-1 (cluster 2), T cells (cluster 3), CD8+ T cells (cluster 4), CD4+ T cells (cluster 5), B cells (cluster 6), DCs (cluster 7) and monocytes-2 (cluster 8) (Fig. 1a,b). More specifically, CD4+ T cells were identified by *CD3G*, *CD4*, *IL2RA* and *IL7R* gene promoter accessibility^10^; CD8+ T cells were identified by *CD3G*, *CD8A* and *CD8B* gene promoter accessibility^11^; NK cells were identified by *CD160*, *GNLY*, *GZMB*, *NKG7* and *KLRF1* gene promoter accessibility^12^; B cells were identified by *CD19*, *CD79A* and *MS4A1* gene promoter accessibility^10,12^; monocytes were identified by *CD14*, *CD68*, *S100A12* and *FCGR1A* gene promoter accessibility^12^; and DCs were identified by *CD83*, *CD1C* and *IL3RA* gene promoter accessibility^10,12,13^ (Fig. 1c,d). Notably, monocyte-1 and monocyte-2 were really different, because we found that the maker gene promoter accessibility of CX3CR1 and CD16 was different in cluster 2 (monocyte-1) and cluster 8 (monocyte-2), which divided monocytes into different subgroups (Supplementary Fig. S2).

Regarding the identified fragments that overlap with the list of TF motifs from the Cell Ranger ATAC pipeline, the most significantly enriched TF motifs in each cluster (Student’s t-test, *p* < 0.01; fold change (FC) > 1.2) with no significant difference between AS_PBMC and NC_PBMC libraries were annotated to show cell-type specificity. Notably, more than 30 TF motifs in monocytes (39 and 48 in monocytes-1 and monocytes-2, respectively) were observed; thus, only the top 10 significantly enriched TF motifs were annotated. As a result, CEBPA, FOS, Nfe2l2, NEUROG2 and SPI1 were critical for monocytes, FOSL2, JUND, CEBPD, CEBPG and FOSL1 were enriched for monocytes-1, and OLIG (OLIG1 and OLIG2), TEAD2, TEDA1 and BHLHE22 were enriched in monocytes-2; GCM1 was increased in T cells, especially CD4+ T cells; CEBPB and CEBPE were abundant in DCs; and TBX20, TBX21, TBX2 and TBR1 were specific to NK cells, which was consistent with data from a previous publication^8^ (Fig. 1e, Supplementary Table S1, Supplementary Spreadsheets S1 and Supplementary Fig. S3).

### Epigenomic analysis of AS_PBMC and NC_PBMC libraries

**Figure 2:**
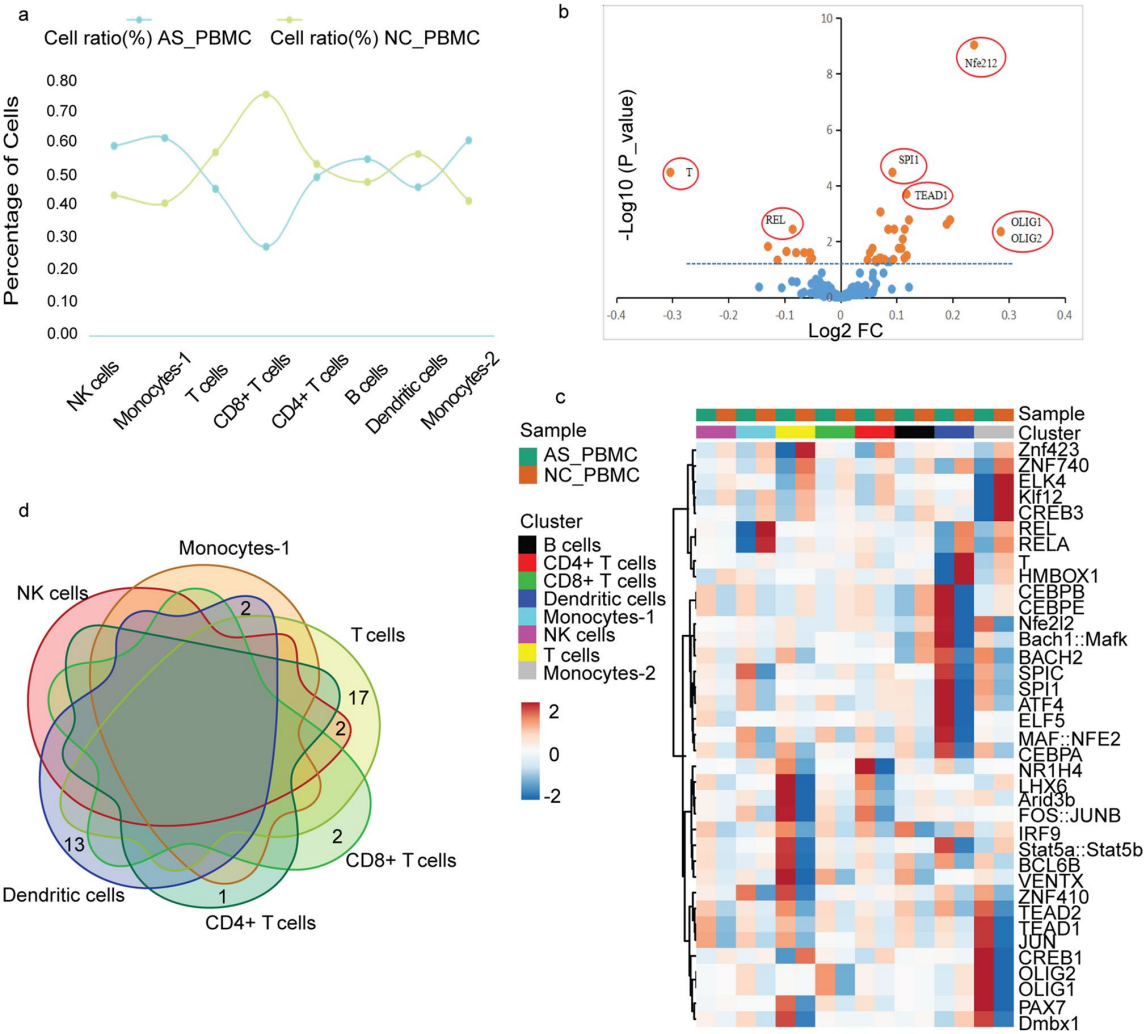
Epigenomic analysis of human PBMCs. (**a**) Percentage of cells in each cell type for comparison of cell number ratio in the AS_PBMC and NC_PBMC libraries; (**b**) Volcano plots of 579 TF motifs in the AS_PBMC library compared to NC_PBMC library; (**c**) Heatmap representation of average counts in the 37 significantly differential TF motifs (rows) across all scATAC-seq clusters from both AS_PBMC and NC_PBMC libraries (columns); (**d**) Venn-diagram showing distribution of 37 significantly differential TF motifs between the AS_PBMC and NC_PBMC libraries. PBMCs, peripheral blood mononuclear cells; AS_PBMC, PBMCs from patients with ankylosing spondylitis (AS); NC_PBMC, PBMCs from healthy controls; NK cells, natural killer cells; TF, transcription factor.

When comparing the 8 clusters in both AS_PBMC and NC_PBMC libraries (Table 2), the cell number in cluster 4 was significantly lower in AS patients (CD8+ T cells, *p* < 0.05, FDR < 0.15) than it was in healthy controls (Fig. 2a), and a total of 37 TF motifs across the genome were significantly different (*p* < 0.05), including 27 more enriched and 10 less active motifs in the AS_PBMC data (Fig. 2b and Supplementary Spreadsheets S2). In detail, 2 different TF motifs (OLIG) were found in CD8+ T cells; among the remaining 7 clusters without significant differences in the cell number ratio, the total number of differential TF motifs in NK cells (TEAD1 and JUN), monocytes-1 (REL and RELA) and CD4+ T cells (NR1H4) was 5; meanwhile, 19 and 15 different TF motifs were found in T cells and DCs, respectively; neither cell numbers nor TF motifs were changed in B cells and monocytes-2.

**Table 2:** Comparison of 8 clusters in both AS_PBMC and NC_PBMC libraries.

Clusters	Cell types	Cell number ratio		*p* value	FDR	Number of differential TF motifs (*p* < 0.05)
		AS	NC			
1	NK cells	28.73	21.25	0.222	1.000	2
2	Monocytes-1	20.42	13.68	0.205	1.000	2
3	T cells	15.16	18.93	0.479	1.000	19
4	CD8+ T cells	6.47	17.59	0.016	0.141	2
5	CD4+ T cells	8.83	9.57	0.857	1.000	1
6	B cells	7.82	6.81	0.784	1.000	0
7	Dendritic cells	6.57	8.04	0.690	1.000	15
8	Monocytes-2	5.99	4.13	0.548	1.000	0

AS_PBMC, peripheral blood mononuclear cells (PBMCs) from AS patients; NC_PBMC, PBMCs from NC; AS, ankylosing spondylitis; NC, healthy controls; *p* value, Student’s t-test; FDR, false discovery rate; TF, transcription factor; NK cells, natural killer cells.

Notably, several TF motifs (such as OLIG) in monocytes-2 from the AS_PBMC group were more accessible than those of the NC_PBMC group, but there were no significant differences (Fig. 2c). Obviously, there were cells sharing the same TF motifs with significant differences (*p* < 0.05) between the AS_PBMC and NC_PBMC libraries (Fig. 2d). In detail, TEAD1 and JUN were more enriched in both NK cells and T cells, while REL and RELA were less accessible in both monocytes-1 cells and DCs.

### Gene regulatory network analysis of the AS_PBMC library

**Figure 3:**
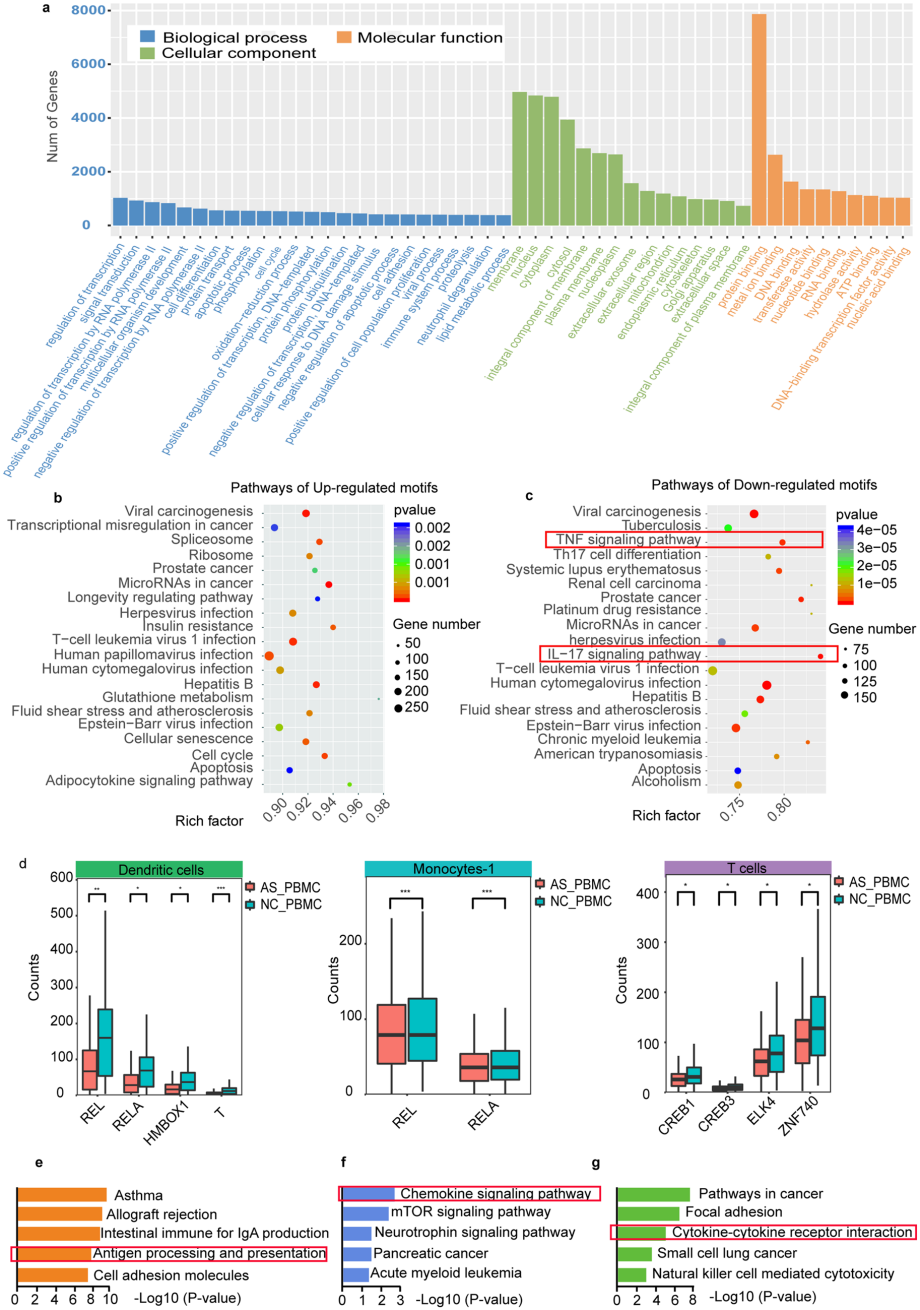
Functional analysis of significantly differential TF motifs between the AS_PBMC and NC_PBMC libraries. (**a**) GO analysis of 12,430 target genes related to the 29 recorded significantly differential TF motifs; KEGG analysis revealing the 20 most significant pathways involved in (**b**) the 21 more accessible TF motifs and (**c**) the 8 less enriched ones from the AS_PBMC group; (**d**) Boxplot showing counts of the 8 less active TF motifs from dendritic cells, monocytes-1 and T cells; Enriched KEGG analysis displaying the 5 most significant pathways related to TF motifs (**e**) OLIG (OLIGI and OLIG2), (**f**) NR1H4, and (**g**) TEAD1 and JUN. *, *p* < 0.05; **, *p* < 0.01, ***p < 0.001, *p*-value was calculated with Loupe Cell Browser 3.1.1 through the difference analysis part, and it was adjusted using the Benjimini-Hochberg correction for multiple tests. TF, transcription factor; PBMCs, peripheral blood mononuclear cells; AS_PBMC, PBMCs from patients with ankylosing spondylitis (AS); NC_PBMC, PBMCs from healthy controls; GO, Gene Ontology; KEGG, Kyoto Encyclopedia of Genes and Genomes.

According to the databases ENCODE, ITFP, Marbach 2016, TRED and TRRUST, 12,430 target genes were found to be related to the significantly differential TF motifs between the AS_PBMC and NC_PBMC libraries, which were validated by ChIP-seq. Since no related genes were ever recorded for TFs of Arid3b, Bach1::Mafk, Dmbx1, Klf12, Nfe2l2, Stat5a::Stat5b, TEAD2 and Znf423, only 29 TFs were used for subsequent Gene Ontology (GO) and Kyoto Encyclopedia of Genes and Genomes (KEGG) analysis. As a result, , 21 TFs could regulate 11,823 target genes with more accessible binding sites in the AS_PBMC group, while the remaining 8 TFs participated in the expression of 8,164 target genes with less accessible binding regions. Based on GO analysis, it was shown that 7868 genes involved in AS pathogenesis had a molecular function of protein binding (Fig. 3a). With further KEGG analysis, the 20 most significant pathways related to both more active TF motifs (Fig. 3b) and less active TF motifs (Fig. 3c) were revealed, and the 8 less enriched TF motifs from the AS_PBMC library were found to play an important role in AS pathogenesis through the IL-17 signaling pathway and TNF signaling pathway (Fig. 3c). Of note, 4 less accessible TF motifs, CREB1, CREB3, ELK4 and ZNF740, were from T cells, and the other 4 less active TF motifs, REL, RELA, T and HMBOX1, were from DCs. Meanwhile, REL and RELA were also less enriched in the monocytes-1 (Fig. 3d and Supplementary Spreadsheets S3).

As mentioned above, the expression of CD8+ T cells was decreased in the AS_PBMC group, while the TF motif OLIG in this cell type was significantly enriched (*p* < 0.05). Based on the databases mentioned above, OLIG was found to be responsible for the transcriptional regulation of 78 and 139 genes, respectively. After deduplication, 142 genes were used for KEGG analysis. 14 genes including CIITA, TAP and HLA-D were found to be abnormally regulated by OLIG2 and they were critical for dysregulated functioning of CD8+ T cells through antigen processing and presentation pathways (Fig. 3e). Noteworthy, CIITA could also be regulated by OLIG1 to participate in the same pathway.

For CD4+ T cells, the TF motif NR1H4 was more accessible in the AS_PBMC group than it was in the NC_PBMC group. With further KEGG analysis, 791 genes could be regulated by NR1H4. Meanwhile, 35genes including ADCY6, ARRB1, BRAF, CCL, CCR, CRK, CXCL, ELMO1, FOXO3, GNB5, GNG, HCK, IKBKG, NFKB1, PF4, PPBP, PREX1, RAP1, RELA, SOS1, SRC, TIAM1 and WAS were identified as able to be regulated to participate in a chemokine signaling pathway (Fig. 3f).

Apart from T cells, monocytes and DCs, NK cells were also found to take part in driving AS progression by regulating 1495 genes with more enriched TF motifs of TEAD1 and JUN. Specifically, TEAD1 was able to regulate 60 genes to be associated with cytokine-cytokine receptor interactions and natural killer cell-mediated cytotoxicity, and JUN could regulate 34 genes to be involved in this pathway (Fig. 3g). Notably, TEAD1 was responsible for the regulation of 1267 genes according to KEGG analysis, while JUN was only involved in the regulation of 297 genes. At the same time, 69 genes were found to be regulated by both TEAD1 and JUN.

## Discussion

Ankylosing spondylitis (AS) is characterized by inflammatory low back pain and progressive ankyloses of spine. This definition has explained that immune cells play a vital role in AS pathogenesis. Since the immune cells circulate in the body through blood, research on PBMCs contributes to finding out markers for diagnosis and target therapy. For example, a recent study on PBMC of AS patients has mentioned that GM-CSF could be detected in plasma from 14/46 (30%) AS patients compared to 3/18 (17%) HC, and GM-CSF neutralization can be a potential novel therapeutic approach for the treatment of AS^14^. In our study, the landscape of active regulatory DNA in PBMCs from AS patients was surveyed at single-cell resolution using the sensitive method of scATAC-seq. Of note, it’s better if the sex ratio between AS patients and HCs is the same^15^. But we used fresh sample in our experiment for higher data quality, and we didn’t obtain samples with the same sex ratio between AS patients and HCs as expected in the hospital. Besides, our study focus on the difference of immune cells between AS patients and the healthy controls instead of cell heterogeneity in AS patients. In addition, there is no significant difference between the result from scATAC-seq of 6 healthy male controls plus 6 female healthy controls and the one from 7 male healthy controls plus 2 female healthy controls. Thus, it is acceptable to have different sex ratio between AS patients and HCs. Based on chromatin accessibility at known marker loci, cells were separated into 8 clusters by type without using antibodies through this method, and T cells, B cells, NK cells, monocytes and DCs were recognized. Notably, two subtypes of monocytes were found. In addition, cell-type-specific TF motifs were identified, such as CEBPA for monocytes, GCM1 for T cells, PRDM1 for B cells, and TBX20 for NK cells, which could be used to identify each cluster in the future.

Since scATAC-seq was able to screen out cells that were involved in the pathogenesis of AS, 8 clusters in both AS_PBMC and NC_PBMC libraries were compared. Consequently, a significant difference in the cell number ratio was observed in CD8+ T cells, suggesting a possible mechanism for AS pathogenesis in which fewer CD8+ T cells fight against inflammation in the AS_PBMC group. Next, all TF motifs among the 8 clusters were analyzed, and 37 differential TF motifs were found. Interestingly, differential TF motifs were hardly observed in the cluster with a significant differential cell number ratio between the AS_PBMC and NC_PBMC libraries, except that TF motif OLIG was more enriched in CD8+ T cells from the AS_PBMC library. Meanwhile, 19 and 15 different TF motifs were found in T cells and DCs, respectively. This result may reveal that most cells take unique actions to promote disease: either they change cell numbers, or they adjust accessibility of their TF motifs. Besides, the 34 (equal to 19 + 15) differential TF motifs may reflect that the normal epigenomic signature of homeostasis for both T cells and DCs was lost in AS patients. In addition, T cells and DCs may contribute to AS progression by altering gene expression. As neither cell numbers nor TF motifs were changed in B cells and monocytes-2, these cells may play a role in AS pathogenesis through other pathways, such as posttranscriptional regulation.

Based on GO and KEGG analysis, 12,430 target genes related to the significantly differential TF motifs between the AS_PBMC and NC_PBMC libraries were found, and 7557 genes were ready for regulation through different TF motifs with opposite changes. This result suggested that these genes may be involved in multiple regulatory networks. Moreover, 7868 genes involved in AS pathogenesis had a molecular function of protein binding and the 8 less enriched TF motifs from the AS_PBMC library were found to play an important role in AS pathogenesis through the IL-17 signaling pathway and TNF signaling pathway, this was in agreement with previous publications^16^.

IL-17 is produced mainly by T cells and is a proinflammatory cytokine. It is involved in AS pathogenesis either by itself or through synergy with other cytokines, such as TNF, to trigger the release of inflammatory mediators and increase the number of immune cells^16^. From our results, all 8 less active TF motifs were found to be critical to the IL-17 signaling pathway because of their regulation of 73 target genes. As the serum concentration of IL-17 was higher in AS patients^16^, our results indicated that the 8 less active TF motifs may contribute to higher expression of the 73 target genes, which resulted in a significant increase in activity of the IL-17 signaling pathway. In fact, the 21 more accessible TF motifs were also involved in the IL-17 signaling pathway, but they were far less statistically significant in their change than the 4 less active ones (*p* value: 0.04 vs 1*10^–7^). Thus, T cells, monocytes-1 and DCs performed their crucial role in the inflammatory response in AS patients by regulating 73 potential target genes through decreasing the accessibility of TF motifs.

Concerning the TNF signaling pathway, TNF was able to recruit receptor-interacting serine/threonine-protein kinase 1 (RIPK1), adaptor protein TNFR1-associated death domain (TRADD), and TNRF-associated factor 2 (TRAF2) to form complex I after binding TNFR1, which could trigger related phosphorylation and ubiquitination processes. Finally, mitogen-activated kinase (MAPK) and nuclear factor kB (NF-kB) were activated to produce proinflammatory effects in AS patients^17^. As a result, 87 target genes were involved in this pathway, and they were regulated by the same 8 TFs with low accessibility that were found in the IL-17 signaling pathway. For the 21 TF motifs that were more accessible in the AS_PBMC group than they were in the NC_PBMC group, only 20 TFs were able to regulate related genes and take part in the TNF signaling pathway. The TF of OLIG1 could be more active in CD8+ T cells from the AS_PBMC group than in CD8+ T cells from the NC_PBMC group, but it was not involved in the TNF signaling pathway. This result may indicate that the binding of OLIG was competitive. Similarly, the significance of KEGG analysis for the 20 more active TF motifs in the AS_PBMC group was much lower than it was for the 8 less accessible TF motifs (*p* value: 0.01 vs 8*10^–7^). Since macrophages, including monocytes and DCs, have been reported to secrete IL-23, which in turn stimulates T cells to produce IL-17 in AS patients^4^, our results confirmed that T cells, monocytes-1 and DCs were very active in AS progression and had diverse roles, such as mediating the IL-17 signaling pathway and TNF signaling pathway to cause inflammation from the perspective of gene transcriptional regulation.

In CD8+ T cells, 14 genes were found to be abnormally regulated and critical for dysregulated cell functioning through antigen processing and presentation pathways. This result was consistent with the report that a particular antigen-specific subset of CD8+ T cells was involved in AS pathogenesis^18^. The TF motif NR1H4 from CD4+ T cells was more accessible in the AS_PBMC group than it was in the NC_PBMC group, and NR1H4 was able to regulate 35 genes to participate in chemokine signaling pathway. As CD4+ T cells mediate AS progression by producing chemokine receptors and cytokines^19^, our results suggested that NR1H4 from CD4+ T cells may be vital for the production of cytokines to cause inflammation. NK cells can recruit other immune cells to produce an excessive immune state by sending activating signals by their receptors in AS^20^. From our results, NK cells were involved in driving AS progression through regulating 60 and 34 genes by more enriched TF motifs of TEAD1 and JUN, respectively, which were associated with cytokine-cytokine receptor interaction and natural killer cell mediated cytotoxicity. This suggests that TEAD1 and JUN may be involved in recruiting other immune cells to produce an excessive immune state. That is, higher accessibility of TEAD1 and JUN could be a signature of malignant NK cells.

In conclusion, T cells, B cells, NK cells, monocytes and DCs were identified without using antibodies. The CD8+ T cell number in AS patients was significantly lower than it was in healthy controls, and 37 differential TF motifs associated with genes responsible for several inflammatory pathways were found. Further, T cells, monocytes-1 and DCs were found to be critical for the IL-17 signaling pathway and TNF signaling pathway through regulating 73 potential target genes, which was the result of 8 TF motifs being less active than they were in healthy controls. Meanwhile, NK cells were able to mediate AS progression by enriching the TF motifs TEAD1 and JUN to regulate 1495 related genes and induce cytokine-cytokine receptor interactions and natural killer cell-mediated cytotoxicity. In addition, CD4+ T cells, as a subset of T cells, may be vital for altering host immune functions by producing cytokines, as the TF motif NR1H4 responsible for the chemokine signaling pathway was more accessible in the AS_PBMC group than it was in the NC_PBMC group. However, for CD8+ T cells, the cell number was lower; on the other hand, TF motif OLIG, which is involved in the pathway of antigen processing and presentation, was more active in the AS_PBMC group than they were in the NC_PBMC group. As for B cells, neither the cell number ratio nor its TF motifs were altered, which suggested that other pathways rather than regulatory elements may play an important role in AS pathogenesis. These findings may explain the core transcriptional circuitry in AS from the perspective of gene transcription regulation by different TF motifs from specific cells, which provides a basic framework to study personal regulomes, and it reveals insights into epigenetic therapy. In the future, differentially expressed genes and proteins can be identified to offer a more theoretical framework for precision medicine. Besides, the reseachers can continue the study based on our results, such as using scATAC-seq to analyze monocyte-1 and find out the most important cells relevant to the disease.

## Methods

### Human PBMC collection

#### Human subjects

Samples were obtained with informed consent in accordance with protocols approved by the ethics committees of both Shenzhen People’s Hospital and Guangzhou Institutes of Biomedicine and Health (Guangzhou, China) (LL-KY-2019363). All AS subjects fulfilling the modified New York criteria^21^ (n = 9, male/female = 7/2, mean age 37 ± 6 years), and healthy controls (n = 12, male/female = 6/6, mean age 34 ± 9 years) were recruited from outpatient clinics or were medical staff in Shenzhen People’s Hospital (Shenzhen, China). No patient was treated with immune suppressants within 3 months of sample collection.

#### Human PBMC collection

Eight milliliters of venous peripheral blood was withdrawn from both patient and control subjects and preserved in heparin tubes. PBMCs were separated by adding equal proportions of Ficoll-Hypaque solution, and then subjected to density-gradient centrifugation (2700 g, 25 min, 25 °C). Next, red blood cell (RBC) lysis buffer was added to remove the remaining RBCs. Finally, PBMCs were washed with chilled PBS, quantified with a cell counting plate and stored on ice for subsequent scATAC-seq analysis.

### scATAC-seq library construction and sequencing

All protocols for performing nuclei isolation and library construction, have been described previously^8^ and are also available here: https://support.10xgenomics.com/single-cell-atac. The important details are as follows:

#### Nuclei isolation

Nuclei suspensions were obtained by incubating lysis buffer (10 mM Tris–HCl, 3 mM MgCl2, 10 mM NaCl , 0.1% Tween-20, 0.1% Nonidet P40 Substitute, 1% BSA) with 1,000,000 cells for 5 min on ice.

#### Library construction

scATAC-seq libraries were generated according to the Chromium Single Cell ATAC protocol (10 × GENOMICS, CG000168) as described previously^8^. Briefly, nuclei suspensions were incubated in a transposition mix that included a transposase and adaptor sequences, which fragmented the DNA in open regions of chromatin; then, barcoded gel beads, transposed nuclei, a master mix, and partitioning oil were loaded on a Chromium Chip E to generate GEMs. Next, silane magnetic beads were used to remove leftover biochemical reagents from the post GEM reaction mixture, and solid-phase reversible immobilization (SPRI) beads were added to eliminate unused barcodes from the samples; after addition of a sample index (P7) and a read 2 (Read 2N) sequence, the final libraries were constructed via PCR with P5 and P7 primers in Illumina bridge amplification. As the Illumina-ready sequencing libraries were produced, Illumina sequencer compatibility, sample indices, sequencing depth and run parameters, library loading and pooling were analyzed.

### scATAC-seq data processing

All protocols for data quality control, genome alignment, peak analysis, clustering and TF motif analysis, have been described previously^8^ and are also available here: https://support.10xgenomics.com/single-cell-atac/software/pipelines/latest/algorithms/overview. The main details are as follows:

#### Barcode processing

To improve data quality, the occasional sequencing error in barcodes obtained from the ‘I2’ index read were fixed. In detail, if barcodes outside the whitelist had Hamming distance less than 2 and their probability of being the real barcodes (based on the abundance in the read data and quality value of the incorrect bases) was more than 90%, they were corrected to whitelist barcodes^8^.

#### Genome alignment

Reference-based analysis was performed using the Cell Ranger ATAC pipeline (https://support.10xgenomics.com/single-cell-atac/software/overview/welcome). First, the adapter and primer oligo sequences were trimmed off. In the current chemistry, a reverse complement of the primer sequence may be found in the 3′ end of a read if the read length was greater than that of the genomic fragment. Thus, the cutadapt tool^22^ was used to identify and trim the reverse complement. Then, BWA-MEM^23^ was applied with default parameters to align the trimmed read pairs that were greater than 25 bp to GRCh38.

#### Duplicate marking

Duplicate reads were found by identifying groups of read pairs across all barcodes, where the 5′ ends of both R1 and R2 have identical mapping positions on the reference. Finally, the unique read pair was reported as a fragment in the file.

#### Peak analysis

As already described^8^, the merged position-sorted BED file was used for peak calling through MACS2^24^. In detail, the number of transposition events at each base pair along the genome was first counted. Next, a smoothed profile of these events with a 401 bp moving window around each base pair and fitting a ZINBA-like mixture model was generated. Then, a signal threshold was set to determine whether a region was a peak signal or noise based on an odds ratio of 1/5. Finally, peaks within 500 bp of each other were to produce a position-sorted BED file. For each barcode, the mapped high-quality fragments that passed all filters were recorded, and the number of fragments that overlapped any peak regions was used to separate the signal from noise. After filtering to contain only cell barcodes, the matrix was used in subsequent analyses, such as dimensionality reduction, clustering and visualization.

#### Clustering and t-SNE projection

Clustering and t-SNE projection were realized by Cell Ranger ATAC pipeline. To cast the data into a lower-dimensional space, dimensionality reduction was first performed via latent semantic analysis (LSA)^25^. The data were normalized via an inverse-document frequency (IDF) transform to provide greater weight to counts in peaks that occurred in rare barcodes. Singular value decomposition (SVD) was performed on this normalized matrix using IRLBA without scaling or centering to produce the transformed matrix in lower-dimensional space. Prior to clustering, normalization to depth was carried out by scaling each barcode data point to the unit L2-norm in the lower dimensional space. Specific to LSA, k-medoid clustering that produced 9 clusters for downstream analysis was provided. In addition, graph-based clustering and visualization via t-SNE were provided^26^, and the data were normalized to unit norms before performing graph-based clustering and t-SNE projection.

#### TF motif identification

Peaks were enriched for transcription factor (TF) binding sites, and the presence of certain motifs was indicative of transcription factor activity. To identify these binding sites, the position weight matrix (PWM) of TF motifs was obtained from the JASPAR database^27^, and each peak was scanned using MOODS (https://github.com/jhkorhonen/MOODS) to find the match for each peak and TF. The threshold *p*-value was 1E-7, and the background nucleotide frequencies were set according to the peak regions in each GC-enriched region. The list of motif-peak matches was unified across these regions, thus avoiding GC bias in the scan.

#### TF motif enrichment analysis

Enrichment of motifs in the peaks was analyzed. First, the reads for a TF motif within a barcode were counted. Then, the ratio of this number to the total read number for that barcode was calculated. Next, the value was normalized to depth. TF motif enrichment was detected by z-scoring the distribution over barcodes of these proportion values. To perform robust analysis, a modified z-score calculation using the median and the scaled median absolute deviation from the median (MAD) was used.

#### Differential accessibility analysis

To find differentially accessible motifs between groups of cells, the fast asymptotic beta test was performed in edgeR by Cell Ranger ATAC pipeline. For each cluster, the algorithm was run on that cluster versus all other cells, yielding a list of genes that were differentially expressed in that cluster relative to the rest of the sample (*p*-value < 0.5). Finally, the relative library size as the total cut site count for each cell divided by the median number was computed.

#### TF motif related genes GO enrichment analysis

Based on the database of ENCODE^28^, ITFP^29^, TRED^30^ and TRRUST^31^, targeted genes related to the specific TF motifs were identified. TF motif-related Gene Ontology (GO) enrichment analysis was performed. First, all peak-related genes were mapped to GO terms in the Gene Ontology database (https://www.geneontology.org/). Then, gene numbers were calculated for each term, and GO terms that were found to be significantly enriched when comparing peak-related genes to the genome background were defined by a hypergeometric test. *p*-values were calculated using the following formula:

$$ P = 1 - \mathop \sum \limits_{i = 0}^{m - 1} \frac{{\left( {\begin{array}{*{20}c} M \\ i \\ \end{array} } \right)\left( {\begin{array}{*{20}c} {N - M} \\ {n - i} \\ \end{array} } \right)}}{{\left( {\begin{array}{*{20}c} N \\ n \\ \end{array} } \right)}} $$

N is the number of all genes with GO annotation; n is the number of peak-related genes in N; M is the number of all genes that are annotated to certain GO terms; and m is the number of peak-related genes in M. The calculated *p*-value was corrected by FDR, and an FDR of less than 0.05 was used as the threshold. GO terms meeting this condition were significantly enriched in peak-related genes. This analysis recognized the main biological functions of related genes.

#### TF motif related genes pathway enrichment analysis

TF motif-related gene pathway enrichment analysis was performed using Kyoto Encyclopedia of Genes and Genomes (KEGG), identifying significantly enriched metabolic pathways and signal transduction pathways in peak-related genes compared to the whole genome background (database: T01001). The *p*-value was calculated using the same equation that was used in GO analysis.

$$ P = 1 - \mathop \sum \limits_{i = 0}^{m - 1} \frac{{\left( {\begin{array}{*{20}c} M \\ i \\ \end{array} } \right)\left( {\begin{array}{*{20}c} {N - M} \\ {n - i} \\ \end{array} } \right)}}{{\left( {\begin{array}{*{20}c} N \\ n \\ \end{array} } \right)}} $$

N is the number of all transcripts with KEGG annotation; n is the number of peak-related genes in N; M is the number of all transcripts annotated to specific pathways; and m is the number of peak-related genes in M.

Notably, Cell Ranger ATAC software was used to perform initial data processing and downstream analysis as described above, while Loupe Cell Browser interactive visualization software was used to generate scATAC-seq peak profiles for cell clusters. *p*-value in this manuscript was calculated with Loupe Cell Browser 3.1.1 through the difference analysis part, and it was adjusted using the Benjimini-Hochberg correction for multiple tests.

#### Deep sequencing data

CBI Gene Expression Omnibus: sequencing data are available under the accession number GSE157595.

## Supplementary information

Supplementary information.

Supplementary information.

Supplementary information.

Supplementary information.

Supplementary information.
