# Supplementary material for: Gene-regulatory network analysis of ankylosing spondylitis with a single-cell chromatin accessible assay
Source: Sci Rep. 2020 Nov 10;10:19411. doi: 10.1038/s41598-020-76574-5 (PMC7655814; doi:10.1038/s41598-020-76574-5)
Supplement: Supplementary file 1 — Supplementary information. [file 41598_2020_76574_MOESM1_ESM.docx]

**Gene-regulatory network analysis of ankylosing spondylitis with a single-cell chromatin accessible assay**

Haiyan Yu^1,2^, Hongwei Wu^3^, Fengping Zheng^1^, Chengxin Zhu^1^, Lianghong Yin^3^, Weier Dai^4^, Dongzhou Liu^1^, Donge Tang^1*^, Xiaoping Hong^1^*, Yong Dai^1^*

^1^ Clinical Medical Research Center, The Second Clinical Medical College of Jinan University (Shenzhen People´s Hospital), Shenzhen, Guangdong 518020, China

^2^Integrated Chinese and Western Medicine Postdoctoral research station, Jinan University, Guangzhou 510632, China

^3^Department of Nephrology, the First Affiliated Hospital of Jinan University, Guangzhou, Guangdong 510632, China

^4^College of Natural Science, University of Texas at Austin, Austin, Texas 78721, United States of America

*Correspondence:

Yong Dai, [daiyong22@aliyun.com](mailto:daiyong22@aliyun.com).

Xiaoping Hong, 1842137378@qq.com.

Donge Tang, [donge66@126.com](mailto:donge66@126.com).


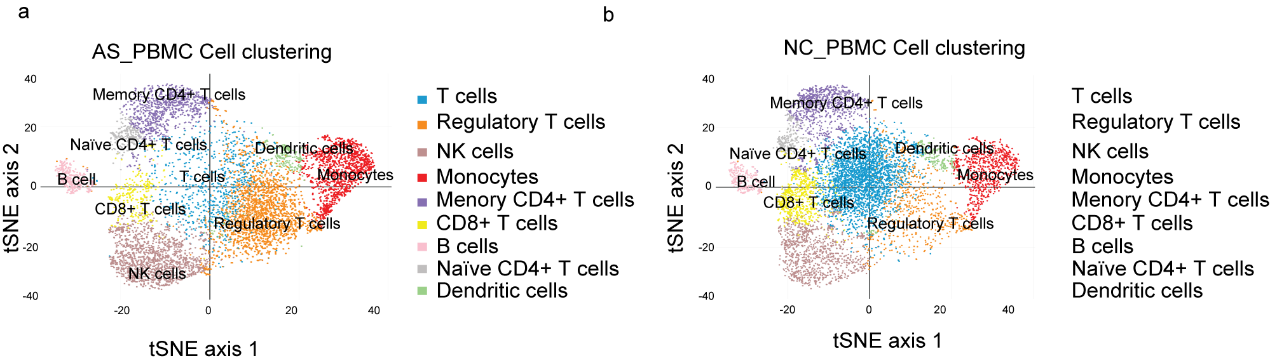


Supplementary Figure 1. Cell-type-specific clustering of human PBMCs according to scATAC-seq. (a) Schematic of cell types in AS_PBMC group; (b) Schematic of cell types in NC_PBMC group. PBMCs, peripheral blood mononuclear cells; scATACseq, assaying transposase-accessible chromatin in single cell sequencing; AS_PBMC, PBMCs from patients with ankylosing spondylitis (AS); NC_PBMC, PBMCs from healthy controls; NK cells, natural killer cells.


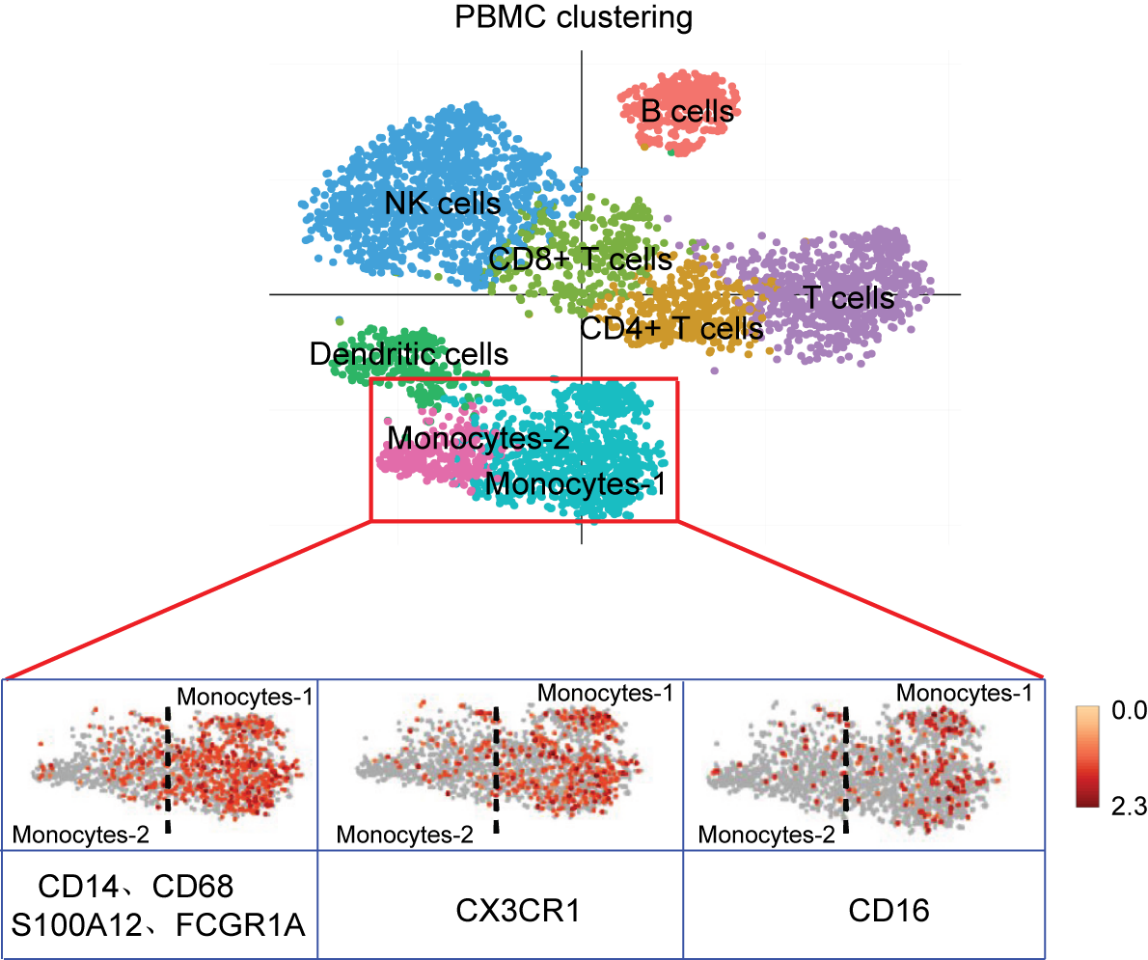


Supplementary Figure 2. tSNE visualization of deviations in accessibility at marker gene promoters.


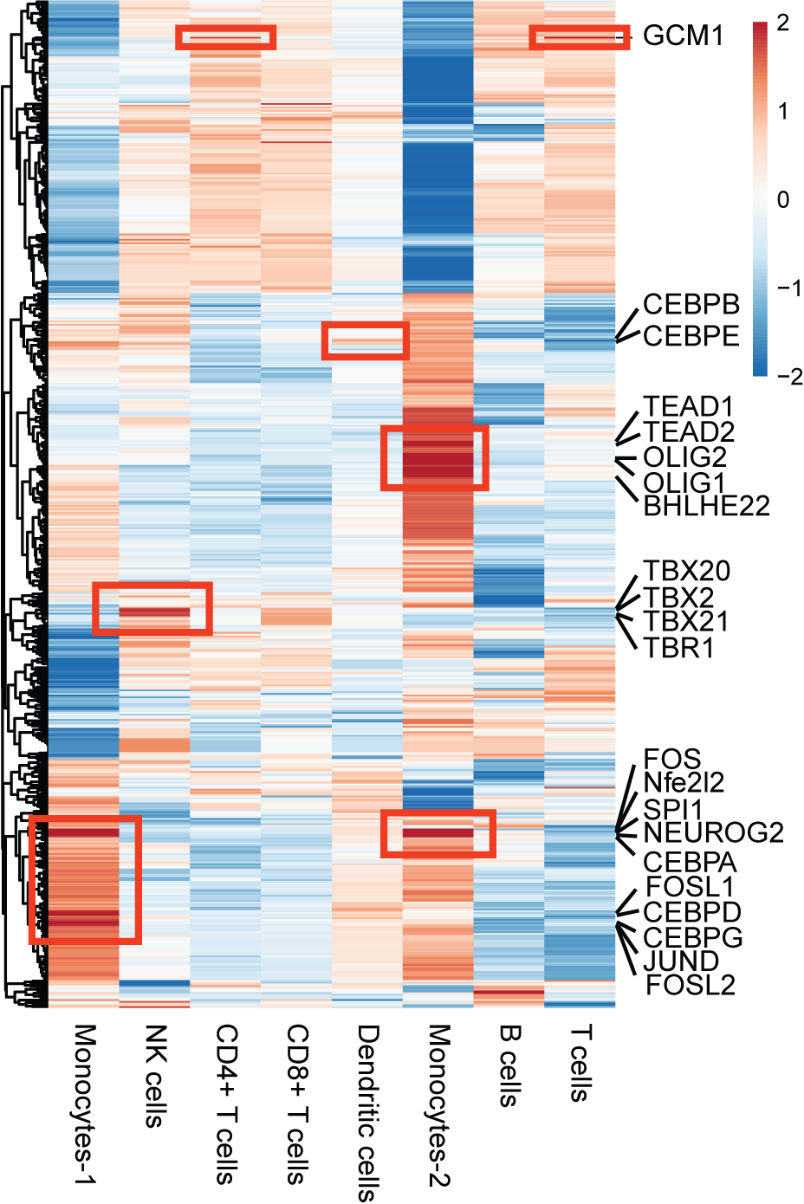


Supplementary Figure 3. Heatmap representation of log 2-fold change in the 579 variable TF motifs (rows) across all scATAC-seq clusters (columns). NK cells, natural killer cells; TF, transcription factor.
