# Supplementary material for: Gene-regulatory network analysis of ankylosing spondylitis with a single-cell chromatin accessible assay
Source: Sci Rep. 2020 Nov 10;10:19411. doi: 10.1038/s41598-020-76574-5 (PMC7655814; doi:10.1038/s41598-020-76574-5)
Supplement: Supplementary file 2 — Supplementary information. [file 41598_2020_76574_MOESM2_ESM.docx]

**Gene-regulatory network analysis of ankylosing spondylitis with a single-cell chromatin accessible assay**

Haiyan Yu^1,2^, Hongwei Wu^3^, Fengping Zheng^1^, Chengxin Zhu^1^, Lianghong Yin^3^, Weier Dai^4^, Dongzhou Liu^1^, Donge Tang^1*^, Xiaoping Hong^1^*, Yong Dai^1^*

^1^ Clinical Medical Research Center, The Second Clinical Medical College of Jinan University (Shenzhen People´s Hospital), Shenzhen, Guangdong 518020, China

^2^Integrated Chinese and Western Medicine Postdoctoral research station, Jinan University, Guangzhou 510632, China

^3^Department of Nephrology, the First Affiliated Hospital of Jinan University, Guangzhou, Guangdong 510632, China

^4^College of Natural Science, University of Texas at Austin, Austin, Texas 78721, United States of America

*Correspondence:

Yong Dai, [daiyong22@aliyun.com](mailto:daiyong22@aliyun.com).

Xiaoping Hong, 1842137378@qq.com.

Donge Tang, [donge66@126.com](mailto:donge66@126.com).

Supplementary Table S1. List of marked transcription factors (TF) motifs included in Figure 1e (FC, Fold Change; 0.000, less than 0.0005).

| TF motifs | TFs | Monocytes-1 | | Monocytes-2 | | Dendritic cells | | Natural killer cells | | CD4+ T cells | | T cells | | |
| --- | --- | --- | --- | --- | --- | --- | --- | --- | --- | --- | --- | --- | --- | --- |
|  |  | FC | P-Value | FC | P-Value | FC | P-Value | FC | P-Value | FC | P-Value | FC | P-Value |  |
| MA0818.1 | BHLHE22 | 1.062 | 0.001 | 1.382 | 0.000 | 1.024 | 0.487 | 0.941 | 0.000 | 0.981 | 0.450 | 1.070 | 0.001 |  |
| MA0102.3 | CEBPA | 1.457 | 0.000 | 1.539 | 0.000 | 1.113 | 0.000 | 0.882 | 0.000 | 0.854 | 0.000 | 0.895 | 0.000 |  |
| MA0466.2 | CEBPB | 1.617 | 0.000 | 1.346 | 0.000 | 1.210 | 0.000 | 0.912 | 0.000 | 0.821 | 0.000 | 0.746 | 0.000 |  |
| MA0836.1 | CEBPD | 1.390 | 0.000 | 1.122 | 0.156 | 1.167 | 0.000 | 0.899 | 0.000 | 0.875 | 0.000 | 0.881 | 0.000 |  |
| MA0837.1 | CEBPE | 1.617 | 0.000 | 1.346 | 0.000 | 1.210 | 0.000 | 0.912 | 0.000 | 0.821 | 0.000 | 0.746 | 0.000 |  |
| MA0838.1 | CEBPG | 1.390 | 0.000 | 1.122 | 0.156 | 1.167 | 0.000 | 0.899 | 0.000 | 0.875 | 0.000 | 0.881 | 0.000 |  |
| MA0476.1 | FOS | 1.426 | 0.000 | 1.479 | 0.000 | 1.083 | 0.000 | 1.027 | 0.007 | 0.866 | 0.000 | 0.730 | 0.000 |  |
| MA0477.1 | FOSL1 | 1.373 | 0.000 | 1.157 | 0.000 | 1.087 | 0.000 | 0.975 | 0.001 | 0.909 | 0.000 | 0.813 | 0.000 |  |
| MA0478.1 | FOSL2 | 1.426 | 0.000 | 1.212 | 0.000 | 1.097 | 0.000 | 0.977 | 0.004 | 0.913 | 0.000 | 0.758 | 0.000 |  |
| MA0646.1 | GCM1 | 0.829 | 0.000 | 0.756 | 0.121 | 0.908 | 0.253 | 0.900 | 0.016 | 1.278 | 0.000 | 1.249 | 0.000 |  |
| MA0491.1 | JUND | 1.395 | 0.000 | 1.187 | 0.000 | 1.101 | 0.000 | 1.006 | 0.528 | 0.902 | 0.000 | 0.744 | 0.000 |  |
| MA0669.1 | NEUROG2 | 1.466 | 0.000 | 1.427 | 0.000 | 1.058 | 0.155 | 0.932 | 0.001 | 0.854 | 0.000 | 0.931 | 0.010 |  |
| MA0150.2 | Nfe2l2 | 1.610 | 0.000 | 1.457 | 0.000 | 1.188 | 0.000 | 0.949 | 0.000 | 0.818 | 0.000 | 0.715 | 0.000 |  |
| MA0826.1/ MA0678.1 | OLIG1/ OLIG2 | 1.068 | 0.015 | 1.659 | 0.000 | 0.990 | 0.874 | 1.031 | 0.214 | 0.887 | 0.000 | 1.006 | 0.851 |  |
| MA0080.4 | SPI1 | 1.559 | 0.000 | 1.369 | 0.000 | 1.143 | 0.000 | 0.832 | 0.000 | 0.842 | 0.000 | 0.852 | 0.000 |  |
| MA0802.1 | TBR1 | 0.719 | 0.000 | 0.854 | 0.001 | 0.894 | 0.000 | 1.306 | 0.000 | 0.971 | 0.087 | 0.831 | 0.000 |  |
| MA0688.1 | TBX2 | 0.831 | 0.000 | 0.934 | 0.083 | 0.941 | 0.000 | 1.254 | 0.000 | 0.944 | 0.000 | 0.842 | 0.000 |  |
| MA0689.1 | TBX20 | 0.822 | 0.000 | 0.993 | 0.998 | 0.932 | 0.007 | 1.364 | 0.000 | 0.881 | 0.000 | 0.700 | 0.000 |  |
| MA0690.1 | TBX21 | 0.871 | 0.000 | 0.807 | 0.000 | 0.933 | 0.006 | 1.298 | 0.000 | 0.918 | 0.000 | 0.752 | 0.000 |  |
| MA0090.2 | TEAD1 | 1.031 | 0.008 | 1.628 | 0.000 | 1.001 | 0.934 | 0.998 | 0.848 | 0.929 | 0.000 | 1.005 | 0.679 |  |
| MA1121.1 | TEAD2 | 1.065 | 0.000 | 1.654 | 0.000 | 0.988 | 0.621 | 0.988 | 0.273 | 0.928 | 0.000 | 1.013 | 0.330 |  |
